# Long-term follow up *Helicobacter Pylori* reinfection rate after second-line treatment: bismuth-containing quadruple therapy versus moxifloxacin-based triple therapy

**DOI:** 10.1186/1471-230X-13-138

**Published:** 2013-09-19

**Authors:** Min Soo Kim, Nayoung Kim, Sung Eun Kim, Hyun Jin Jo, Cheol Min Shin, Young Soo Park, Dong Ho Lee

**Affiliations:** 1 Department of Internal Medicine, Seoul National University Bundang Hospital, Seongnam, Gyeonggi-do, South Korea; 2 Department of Internal Medicine and Liver Research Institute, Seoul National University College of Medicine, Seoul, South Korea

**Keywords:** *Helicobacter pylori*, Reinfection, Quadruple, Moxifloxacin, Second-line

## Abstract

**Background:**

The increasing trend of antibiotic resistance requires effective second-line *Helicobacter pylori* (*H. pylori*) treatment in high prevalence area of *H. pylori.* The aim of our study was to evaluate the reinfection rate of *H. pylori* after second-line treatment that would determine the long-term follow up effect of the rescue therapy.

**Methods:**

A total of 648 patients who had failed previous *H. pylori* eradication on standard triple therapy were randomized into two regimens: 1, esomeprazole (20 mg b.i.d), tripotassium dicitrate bismuthate (300 mg q.i.d), metronidazole (500 mg t.i.d), and tetracycline (500 mg q.i.d) (EBMT) or 2, moxifloxacin (400 mg q.d.), esomeprazole (20 mg b.i.d), and amoxicillin (1000 mg b.i.d.) (MEA). At four weeks after completion of eradication therapy, *H. pylori* tests were performed with ^13^C urea breath test or invasive tests. In patients who maintained continuous *H. pylori* negativity for the first year after eradication therapy, *H. pylori* status was assessed every year. For the evaluation of risk factors of reinfection, gender, age, clinical diagnosis, histological atrophic gastritis or intestinal metaplasia were analyzed.

**Results:**

The recrudescence rate of the EBMT was 1.7% and of the MEA group 3.3% (*p* = 0.67). The annual reinfection rate of *H. pylori* of EBMT was found to be 4.45% and the MEA group 6.46%. Univariate analysis (Log-rank test) showed no association with any clinical risk factor for reinfection.

**Conclusions:**

The long-term reinfection rate of *H. pylori* stayed low in both of bismuth-containing quadruple therapy and moxifloxacin-based triple therapy; thus reinfection cannot affect the choice of second-line treatment.

**Trial registration:**

Clinical Trial Registration Number NCT01792700

## Background

Helicobacter *pylori* (*H. pylori*) is a common pathogen of the gastric mucosa. It is estimated that at least 50% of the world’s human population has *H. pylori* infection [1]. Since the majority of patients with *H. pylori* infection do not have any related clinical disease, routine screening is not considered [2]. However, as the current evidence suggests that *H. pylori* play a major role in peptic ulcer disease, gastric MALT lymphoma and in gastric cancer [3], screening and treatment in these diseases are recommended in several guidelines [2,4-7]. In addition, European guidelines recommend eradicating *H. pylori* infection in first-degree relatives of patients with gastric cancer, in long term NSAIDS or acid suppression users and in patients with functional dyspepsia [4]. According to these guidelines, public health efforts toward eradication will be more effective in *H. pylori* high prevalence areas. Naturally, it is expected that increasing use of antibiotics must lead to increased resistance of antibiotics. Currently, the most commonly used initial treatment is a triple regimen combining a proton pump inhibitor (PPI) with two antibiotics (clarithromycin and amoxicillin/or metronidazole) for the eradication of *H. pylori*[2,4-7]. Although this regimen has been shown to be effective in numerous clinical trials, the most recent data show that the eradication rate has declined to less than 80% worldwide, largely related to development of resistance to clarithromycin [8]. In Korea, the recent eradication rate of this regimen was less than 80% in a long-term follow up study (≥ 5 years) [9,10]. Therefore, this decreasing eradication rate requires effective second-line treatment. Many clinicians have been using second-line therapy with bismuth-containing quadruple therapy or including fluoroquinolone antibiotics such as levofloxacin and moxifloxacin. In this situation, reinfection of *H. pylori* will determine the long-term effect of the eradication therapy for *H. pylori*. If a regimen shows a high reinfection rate, then this eradication therapy should be avoided or strictly used only when absolutely indicated for *H. pylori* eradication. We reported the long-term annual reinfection rate of *H. pylori* in standard PPI-based triple therapy to be 3.51% per year in Korea [11]. Now that second-line therapy is frequently used there is increasing interest regarding the reinfection and recrudescence rates after rescue therapy. However, there are few reports regarding the reinfection rate of *H. pylori* after quadruple therapy [12] and none for quinolone based triple therapy. From this background the aim of our study was to evaluate the reinfection rate of *H. pylori* after two kinds of second-line treatment over a long-term follow up period. In addition, we investigated the risk factors for reinfection after this second-line treatment.

## Methods

### Study population

**Figure 1:**
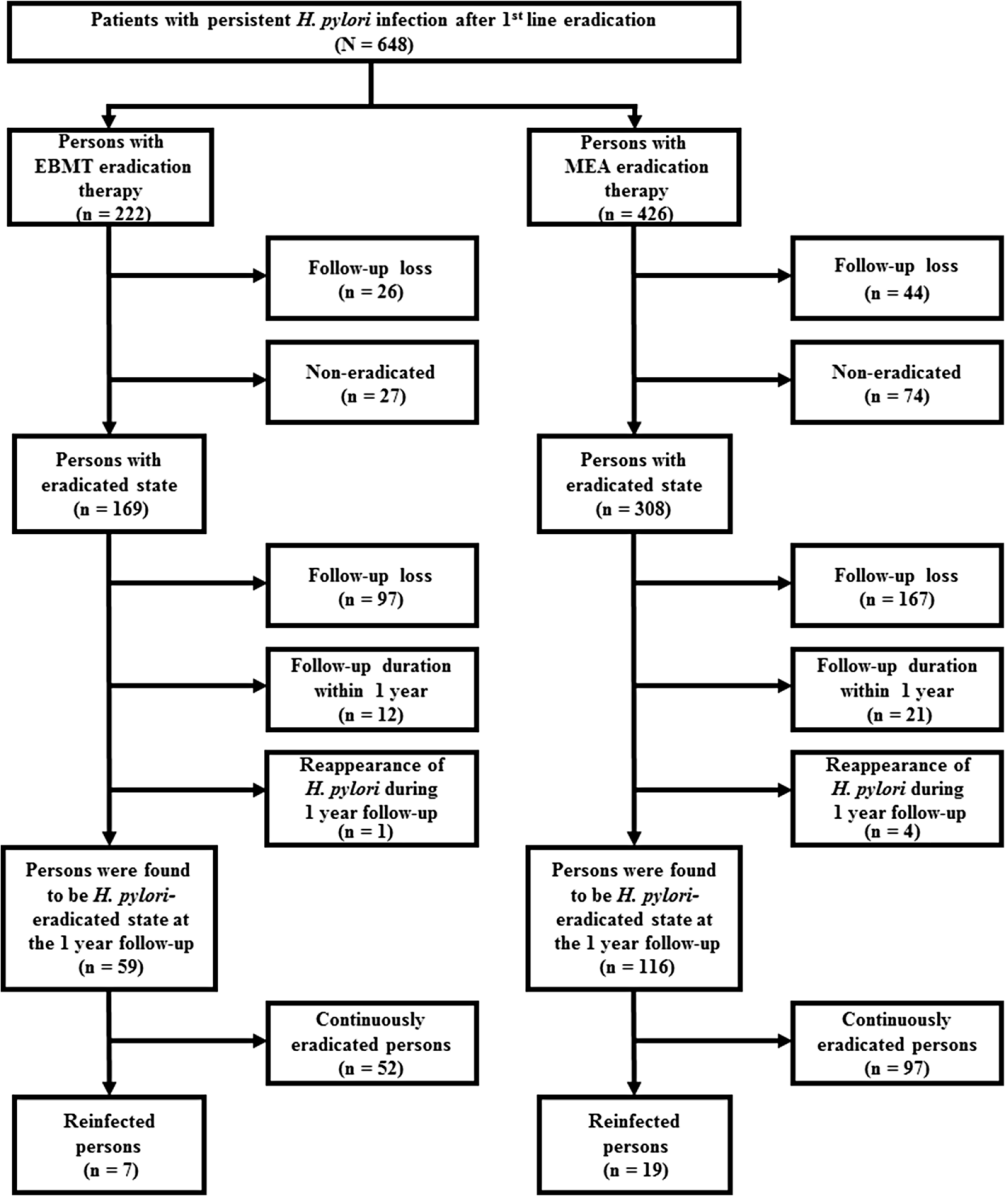
Schematic study flow chart.

The schematic flow of this study is shown in Figure 1. This was a prospective study performed between 2003 and 2010 at Seoul National University Bundang Hospital in Korea. A total of 648 patients with persistent *H. pylori* infection after first-line treatment (PPI-based triple therapy) were enrolled. PPI-based triple therapy included PPI (standard dose), amoxicillin 1 g, and clarithromycin 500 mg, all twice daily, for 7 days. Patients were considered persistent *H. pylori* infection if ^13^C-urea breath test (UBT) or invasive *H. pylori* test (Giemsa histology, CLO test, culture) were positive despite PPI-based triple therapy. Patients were excluded from the study if they had a history of renal or hepatic impairment, previous gastric surgery, pregnancy or lactation, therapy with steroids or non-steroidal anti-inflammatory drugs, or therapy with a proton pump inhibitor (PPI) or antibiotics within four weeks of entry. Between 2003 and 2006, the 44 patients with persistent *H. pylori* infection were treated with bismuth-containing quadruple therapy. Between 2007 and 2010, 604 patients with persistent *H. pylori* infection were randomized into two kinds of second-line therapy (bismuth-containing quadruple therapy or moxifloxacin-based triple therapy). However, if the patient preferred one regimen, after sufficient information for side effect and eradication rate of each regimen a change was permitted. Finally, 222 patients were treated for 14 days with esomeprazole 20 mg b.i.d, tripotassium dicitrate bismuthate 300 mg q.i.d, metronidazole 500 mg t.i.d, and tetracycline 500 mg q.i.d (EBMT) as second-line treatment regimen for *H. pylori* infection. 426 patients were treated for 14 days with moxifloxacin 400 mg q.d, esomeprazole 20 mg b.i.d, and amoxicillin 1000 mg b.i.d (MEA) as second-line treatment regimen for *H. pylori* infection. At four weeks after completion of the second-line treatment, *H. pylori* eradication was evaluated by ^13^C-UBT or invasive tests. Invasive tests were performed in the patients in whom follow up endoscopic examination was necessary for peptic ulcer, adenoma or gastric cancer. *H. pylori* negative status after eradication was defined as a negative ^13^C-UBT or all negative of Giemsa stain, CLO test, and culture. Among 222 patients with EBMT eradication therapy and 426 patients with MEA eradication therapy, 169 patients and 308 patients were found to be in eradicated status, respectively (Figure 1).

All subjects provided informed consent, and the study protocol was approved by the Ethical Committee at Seoul National University Bundang Hospital. ClinicalTrials.gov registration number is NCT01792700.

### Invasive *Helicobacter pylori* test (Giemsa histology, CLO test, and culture) and histology

To determine the presence of current *H. pylori* infection, 10 biopsy specimens were taken from the gastric mucosa at each endoscopy (two biopsy specimens each from the greater curvature of the antrum and body, and three each from the lesser curvature of the antrum and body). Among them, four biopsy specimens (one each from the greater curvature and lesser curvature of the antrum and body) were fixed in formalin, and used for determination of *H. pylori* infection by Giemsa staining. Another four specimens from the four gastric mucosa areas mentioned above were used for *H. pylori* culturing. The remaining two specimens from the lesser curvature of the antrum and body were used for the rapid urease test (CLO test; Delta West, Bentley, Australia).

Four of the biopsy specimens used for determination of *H. pylori* infection were also used for histological evaluation. These specimens were examined for the presence of gastric atrophy and intestinal metaplasia by H&E staining. The presence of atrophy on any of four specimens was diagnosed as gastric atrophy, and the same method was applied to intestinal metaplasia. The definition of atrophy is the loss of appropriate glands including both metaplastic and non-metaplastic atrophy. Both metaplastic and non-metaplastic atrophy can be allocated to one of three grades of severity using grading criteria modeled on those suggested by the original and the updated Sydney System [13].

### ^13^C-urea breath test

Patients fasted for 4 h before testing. Then, 100 mg of ^13^C-urea powder (UBiTkit; Otsuka Pharmaceutical, Tokyo, Japan) was dissolved in 100 mL water and administered orally; a second breath sample was collected 20 min later. The collected samples were analyzed using an isotope-selective, non-dispersive infrared spectrometer (UBiT-IR300; Otsuka Pharmaceutical). The cutoff value used for *H. pylori* eradication was 2.5‰.

### Follow-up of *H. pylori* tests

All of the eradicated patients received gastroscopy with invasive tests (modified Giemsa stain and CLO test) not only from greater and lesser curvature of antrum but also from body after 1 year. If any one of these tests were positive then the patient was regarded as recrudescence case. After this time the patients were followed up for one year with gastroscopy with invasive tests. However, when the patients preferred ^13^C**-**UBT or wanted to receive the gastroscopy every other year it was also accepted because the Korea government national health insurance program recommends biannual endoscopy instead of one year.

### Statistical analysis

The annual reinfection rate (percentage per year) of *H. pylori* was calculated as (total number of infected patients/ cumulative observation years for all patients) X 100.

SPSS for Windows (version 18.0; SPSS, Inc., an IBM Company, Chicago, Illinois, USA) was used for all statistical analyses. Categorical variables were analyzed using the Pearson chi-square test or Fisher’s exact test, and continuous variables were analyzed using independent samples *t*-test. The risk of *H. pylori* reinfection with time was estimated using the Kaplan-Meier method. To determine the risk factors for reinfection, we used the log-rank test. Null hypotheses of no difference were rejected if *p*-values were less than 0.05.

## Results

### Patient characteristics

Among eradicated 169 patients in the EBMT group and eradicated 308 patients in the MEA group, 59 patients and 116 patients maintained *H. pylori*-negative status continuously for one year, respectively (Figure 1). Specifically, 110 patients dropped out in the EBMT group and 192 in the MEA group for the following three reasons: 97 patients in the EBMT group and 167 in the MEA group for not returning for gastroscopy or ^13^C**-**UBT after treatment, 12 in the EBMT group and 21 in the MEA group for follow-up duration within 1 year, 1 patient in the EBMT group and 4 in the MEA group for reappearance of *H. pylori* during 1 year follow-up. Finally, 59 patients and 116 patients in each group maintained *H. pylori*-negative continuously at one year. The demographic and clinical characteristics of two study groups, who maintained *H. pylori*-negative continuously at one year after the EBMT or MEA therapy, are summarized in Table 1. Gender, the mean age of the patients, clinical diagnosis, atrophic gastritis, and intestinal metaplasia of the two groups were similar. The enrolled early gastric cancer patients were cured by endoscopic submucosal dissection and follow-up was continuously performed regularly. During long-term follow-up patients in the EBMT or in the MEA group were divided into two groups: reinfected group and continuously eradicated group. The demographic and clinical characteristics of the reinfected and continuously eradicated group are summarized in Table 2. In the EBMT group and MEA group, there was no significant evidence that reinfection of *H. pylori* was related with gender, the mean age of the patients, clinical diagnosis, atrophic gastritis, and intestinal metaplasia. The *H. pylori* recrudescence and reinfection rates are shown in Table 3. One patient in the EBMT group and four patients in the MEA group, who were *H. pylori* positive again at 1 year follow-up, were assigned to recrudescence cases. The rate was calculated at 1.7% (1/60) for the EBMT group and 3.3% (4/120) for the MEA group, and these percentages were not significantly different (*p* = 0.67). During long-term follow-up 1 year after eradication *H. pylori* reappeared in 7 (11.9%) of EBMT group and in 19 (16.4%) of MEA group and these percentages were not significantly different depending on each rescue treatment (*p* = 0.43). Among the reinfected persons no one was belonged to the same household.

**Table 1:** Baseline demographic and clinical characteristics of subjects who maintained the eradicated state by quadruple therapy (EBMT) or moxifloxacin-based triple therapy (MEA)

**Variable category**	**Total**	**EBMT**	**MEA**	*p*-value^*^
	**(N = 175)**	**(N = 59)**	**(N = 116)**	
**Gender**	**(N = 175)**	**(n = 59)**	**(n = 116)**	
Male	(104, 59.4%)	(35, 59.3%)	(69, 59.5%)	0.98
Female	(71, 40.6%)	(24, 40.7%)	(47, 40.5%)	
**Age (years) (mean ± SD)**	**(N = 175)**	**(n = 59)**	**(n = 116)**	0.67
	(56.6 ± 9.4)	(56.1 ± 9.3)	(56.8 ± 9.5)	
**Clinical diagnosis**	**(N = 175)**	**(n = 59)**	**(n = 116)**	
Early gastric cancer	(40, 22.9%)	(11, 18.6%)	(29, 25.0%)	0.06
Dysplasia	(19, 10.9%)	(3, 5.1%)	(16, 13.8%)	
Peptic ulcer disease	(34, 19.4%)	(17, 28.8%)	(17, 14.7%)	
Chronic gastritis	(82, 46.9%)	(28, 47.5%)	(54, 46.6%)	
**Histological AG in either antrum or body**	**(N = 116)**	**(n = 37)**	**(n = 79)**	
Yes	(67, 57.8%)	(21, 56.8%)	(46, 68.7%)	0.88
No	(49, 42.2%)	(16, 43.2%)	(33, 41.8%)	
**Histological IM in either antrum or body**	**(N = 144)**	**(n = 47)**	**(n = 97)**	
Yes	(84, 58.3%)	(23, 48.9%)	(61, 62.9%)	0.11
No	(60, 41.7%)	(24, 51.1%)	(36, 37.1%)	

*EBMT*: esomeprazole (20 mg b.i.d), tripotassium dicitrate bismuthate (300 mg q.i.d), metronidazole (500 mg t.i.d), and tetracycline (500 mg q.i.d); *MEA*: moxifloxacin (400 mg q.d.), esomeprazole (20 mg b.i.d), and amoxicillin (1000 mg b.i.d.); *AG*, atrophic gastritis, *IM*, intestinal metaplasia.

All of early gastric cancer patients were cured by endoscopic submucosal dissection.

*p-value for Pearson chi-square test for comparison of categorical data, and independent samples *t*-test for comparison of age.

**Table 2:** Baseline demographic and clinical characteristics of study subjects depending on reinfection

	**EBMT**				**MEA**			
**Variable category**	**Total (59, 100%)**	**Reinfected group (7, 11.9%)**	**Continuously eradicated group (52, 88.1%)**	*p*-value^*^	**Total (116, 100%)**	**Reinfected group (19, 16.4%)**	**Continuously eradicated group (97, 83.6%)**	*p*-value^*^
**Gender**	(N = 59)	(n = 7)	(n = 52)		(N = 116)	(n = 19)	(n = 97)	
Male	(35, 59.3%)	(4, 57.1%)	(31, 59.6%)	0.816	(69, 59.5%)	(12, 63.2%)	(57, 58.8%)	0.353
Female	(24, 40.7%)	(3, 42.9%)	(21, 40.4%)		(47, 40.5%)	(7, 36.8%)	(40, 41.2%)	
**Age (mean ± SD)**	(N = 59)	(n = 7)	(n = 52)		(N = 116)	(n = 19)	(n = 97)	
	(56.1 ± 9.3)	(59.9 ± 9.8)	(55.6 ± 9.2)		(56.8 ± 9.5)	(56.7 ± 9.4)	(56.8 ± 9.6)	
20-29	(1, 1.7%)	(0, 0.0%)	(1, 1.9%)	0.306	(1, 0.9%)	(0, 0.0%)	(1, 1.0%)	0.479
30-39	(1, 1.7%)	(0, 0.0%)	(1, 1.9%)		(3, 2.6%)	(0, 0.0%)	(3, 3.1%)	
40-49	(13, 22.0%)	(2, 22.2%)	(11, 21.2%)		(21, 18.1%)	(6, 31.6%)	(15, 15.5%)	
50-59	(21, 35.6%)	(1, 14.3%)	(20, 38.5%)		(40, 34.5%)	(5, 26.3%)	(35, 36.1%)	
60-69	(20, 33.9%)	(3, 42.9%)	(17, 32.7%)		(42, 36.2%)	(6, 31.6%)	(36, 37.1%)	
70-79	(3, 5.1%)	(1, 14.3%)	(2, 3.8%)		(8, 10.5%)	(2, 10.5%)	(6, 6.2%)	
80-89	(0, 0.0%)	(0, 0.0%)	(0, 0.0%)		(1, 0.6%)	(0, 0.0%)	(1, 1.0%)	
**Clinical diagnosis**	(N = 59)	(n = 7)	(n = 52)		(N = 116)	(n = 19)	(n = 97)	
Early gastric cancer	(11, 18.6%)	(0, 0.0%)	(11, 21.2%)	0.198	(29, 25.0%)	(7, 36.8%)	(22, 22.7%)	0.77
Dysplasia	(3, 5.1%)	(1, 14.3%)	(2, 3.8%)		(16, 13.8%)	(3, 15.8%)	(13, 13.4%)	
Peptic ulcer disease	(17, 28.8%)	(4, 57.1%)	(13, 25.0%)		(17, 14.7%)	(3, 15.8%)	(14, 14.4%)	
Chronic gastritis	(28, 47.5%)	(2, 28.6%)	(26, 50.0%)		(54, 46.6%)	(6, 31.6%)	(48, 49.5%)	
**Histological AG in either antrum or body**	(N = 37)	(n =6)	(n = 31)		(N = 79)	(n = 14)	(n = 65)	
Yes	(21, 56.8%)	(5, 83.3%)	(16, 51.6%)	0.113	(46, 58.2%)	(9, 64.3%)	(37, 56.9%)	0.575
No	(16, 43.2%)	(1, 16.7%)	(15, 48.4%)		(33, 41.8%)	(5, 35.7%)	(28, 43.1%)	
**Histological IM in either antrum or body**	(N = 47)	(n = 6)	(n = 41)		(N = 97)	(n = 15)	(n = 82)	
Yes	(23, 48.9%)	(4, 66.7%)	(21, 51.2%)	0.193	(61, 62.9%)	(8, 53.3%)	(53, 64.6%)	0.52
No	(24, 51.1%)	(2, 33.3%)	(20, 48.8%)		(36, 37.1%)	(7, 46.7%)	(29, 35.4%)	

*EBMT*: esomeprazole (20 mg b.i.d), tripotassium dicitrate bismuthate (300 mg q.i.d), metronidazole (500 mg t.i.d), and tetracycline (500 mg q.i.d); *MEA*: moxifloxacin (400 mg q.d.), esomeprazole (20 mg b.i.d), and amoxicillin (1000 mg b.i.d.); *AG*, atrophic gastritis; *IM*, intestinal metaplasia.

All of early gastric cancer patients were cured by endoscopic submucosal dissection.

^*^ P-value for Log-rank test.

**Table 3:** **Reinfection and recrudescence rate of *Helicobacter pylori***

**Variable category**	**E B M T group**	**M E A group**	*p*-value^*^
**Recrudescence**	(n = 60)	(n = 120)	
Yes	1 (1.7%)	4 (3.3%)	0.67
No	59 (98.3%)	116 (96.7%)	
**Reinfection**	(n = 59)	(n = 116)	
Yes	7 (11.9%)	19 (16.4%)	0.43
No	52 (88.1%)	97 (83.6%)	

*EBMT*: esomeprazole (20 mg b.i.d), tripotassium dicitrate bismuthate (300 mg q.i.d), metronidazole (500 mg t.i.d), and tetracycline (500 mg q.i.d); *MEA*: moxifloxacin (400 mg q.d.), esomeprazole (20 mg b.i.d), and amoxicillin (1000 mg b.i.d.).

* P-value for Pearson chi-square test or Fisher’s exact test for comparison of categorical data.

### Long-term follow-up and reinfection rate

The mean duration of follow-up of 59 patients in the EBMT group and 116 in the MEA group was 31.9 months (range: 18–90 months) and 30.4 months (range: 18–59 months). The mean number of *H. pylori* tests per patient was found to be 2.05 tests for the EBMT group and 2.31 tests for the MEA group (Table 4). Reinfection with *H. pylori* occurred in 7 of 59 patients of EBMT group (11.9%) and in 19 of 116 patients of MEA group (16.4%) sporadically during the follow-up period. The calculated total annual reinfection rate was found to be 4.45% (7/157.17 patient years X 100) for EBMT and 6.46% (19/294.08 patient years X 100) for MEA.

**Table 4:** **Annual reinfection rate of *Helicobacter pylori***

	**Follow up period**	**No. of patients**	**Mean no. of *H. pylori *test**	**No. of reinfected patients**	**Patient-years (yr)**	**Annual reinfection rate (%)**
	1 ≤ year <2	23	1.39	3	33.08	9.07
**E**	2 ≤ year <3	18	2.22	3	42.25	7.10
**B**	3 ≤ year <4	5	2.00	0	16.58	0
**M**	4 ≤ year <5	8	2.63	0	34.67	0
**T**	5 ≤ year <6	3	6.00	1	16.58	6.03
	6 ≤ year <7	1	5.00	0	6.5	0
	7 ≤ year <8	1	5.00	0	7.5	0
	**Total**	**59**	**2.05**	**7**	**157.17**	**4.45**
	1 ≤ year <2	39	1.36	8	54.12	14.78
**M**	2 ≤ year <3	37	2.05	8	91.75	8.72
**E**	3 ≤ year <4	30	3.67	1	104.58	0.96
**A**	4 ≤ year <5	10	3.80	2	43.33	4.62
	**Total**	**116**	**2.31**	**19**	**294.08**	**6.46**

*EBMT*: esomeprazole (20 mg b.i.d), tripotassium dicitrate bismuthate (300 mg q.i.d), metronidazole (500 mg t.i.d), and tetracycline (500 mg q.i.d); *MEA*: moxifloxacin (400 mg q.d.), esomeprazole (20 mg b.i.d), and amoxicillin (1000 mg b.i.d.).

### Risk factors for reinfection

**Figure 2:**
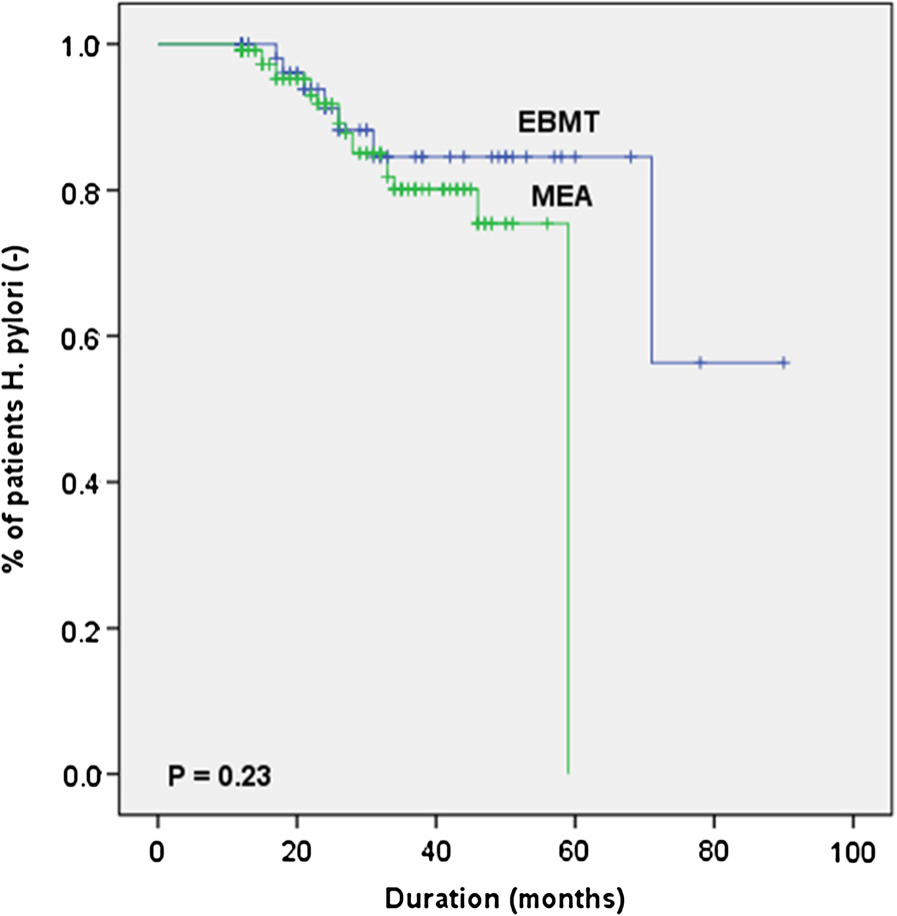
**Kaplan-Meier curves for *Helicobacter pylori *reinfection according to regimen.**

When the reinfected group (n = 26) and continuously eradicated group (n = 149) were compared in terms of demographic information and clinical characteristics, no statistical differences were found by univariate analysis (Log-rank test), in both groups (Table 5). Specially, there was no significant evidence that reinfection of *H. pylori* is related with eradication regimen (*p* = 0.23) (Figure 2).

**Table 5:** Baseline characteristics of study subjects

**Variable category**	**Total (175, 100%)**	**Reinfected group (26, 14.9%)**	**Continuously eradicated group (149, 85.1%)**	*p*-value^*^
**Gender**	**(N = 175)**	**(n = 26)**	**(n = 149)**	
Male	(104, 59.4%)	(16, 61.5%)	(88, 59.1%)	0.75
Female	(71, 40.6%)	(10, 38.5%)	(61, 40.9%)	
**Age (years) (mean ± SD)**	**(N = 175)**	**(n = 26)**	**(n = 149)**	
	**(56.6 ± 9.4)**	**(57.6 ± 9.4)**	**(56.4 ± 9.5)**	
20-29	(2, 1.1%)	(0, 0.0%)	(2, 1.3%)	0.47
30-39	(4, 2.3%)	(0, 0.0%)	(4, 2.7%)	
40-49	(34, 19.4%)	(8, 30.8%)	(26, 17.4%)	
50-59	(61, 34.9%)	(6, 23.1%)	(55, 36.9%)	
60-69	(62, 35.4%)	(9, 34.6%)	(53, 35.6%)	
70-79	(11, 6.3%)	(3, 11.5%)	(8, 5.4%)	
80-89	(1, 0.6%)	(0, 0.0%)	(1, 0.7%)	
**Clinical diagnosis**	**(N = 175)**	**(n = 26)**	**(n = 149)**	
Early gastric cancer	(40, 22.9%)	(7, 26.9%)	(33, 22.1%)	0.74
Dysplasia	(19, 10.9%)	(4, 15.4%)	(15, 10.1%)	
Peptic ulcer disease	(34, 19.4%)	(7, 26.9%)	(27, 18.1%)	
Chronic gastritis	(82, 46.9%)	(8, 30.8%)	(74, 49.7%)	
**Histological AG in either antrum or body**	**(N = 116)**	**(n = 20)**	**(n = 96)**	
Yes	(67, 57.8%)	(14, 70.0%)	(53, 55.2%)	0.14
No	(49, 42.2%)	(6, 30.0%)	(43, 44.8%)	
**Histological IM in either antrum or body**	**(N = 144)**	**(n = 21)**	**(n = 123)**	
Yes	(84, 58.3%)	(10, 47.6%)	(74, 60.2%)	0.20
No	(60, 41.7%)	(11, 52.4%)	(49, 39.8%)	
**Regimen**	**(N = 175)**	**(n = 26)**	**(n = 149)**	
EBMT	(59, 33.7%)	(7, 26.9%)	(52, 34.9%)	0.23
MEA	(116, 66.3%)	(19, 73.1%)	(97, 65.1%)	

*EBMT*: esomeprazole (20 mg b.i.d), tripotassium dicitrate bismuthate (300 mg q.i.d), metronidazole (500 mg t.i.d), and tetracycline (500 mg q.i.d); *MEA*: moxifloxacin (400 mg q.d.), esomeprazole (20 mg b.i.d), and amoxicillin (1000 mg b.i.d.); *AG*, atrophic gastritis; *IM*, intestinal metaplasia.

All of early gastric cancer patients were cured by endoscopic submucosal dissection.

^*^ P-value for Log-rank test.

## Discussion

We performed a prospective study to investigate reinfection rate of *H. pylori* in patients who had been successfully treated with second-line therapy after an initial failure to eradicate *H. pylori*. To the best of our knowledge, this is the first report comparing the reinfection rate of EBMT and MEA therapy.

Reinfection is defined as an infection with a new strain of *H. pylori* that is different from the original strain after complete eradication, while recrudescence is a relapse of original strain, which was temporarily suppressed by eradication therapy [14,15]. The recurrence rates of *H. pylori* decrease with time and decline sharply after the first year, and beyond the first year, recurrence rates come close to the rate of natural acquisition of *H. pylori* infection in adulthood [14,16,17]. From these reports the confirmation of continuous *H. pylori* negativity for the first year after eradication therapy has been accepted as complete eradication [18-20]. Therefore, in our study, reinfection was defined as the situation where tests for *H. pylori* infection, after continuous *H. pylori* negativity for the first year after eradication therapy, become positive again at a later stage. In addition, patients, who become *H. pylori* positive again during 1 year follow-up were classified as recrudescence cases. We could not perfectly distinguish between recrudescence of an original strain and reinfection because DNA analysis of the strain using molecular fingerprinting techniques was not performed. However, this definition is supported by data obtained using DNA analysis that the cause of *H. pylori* recurrence after first year is reinfection [14].

In the previous study, we reported that annual reinfection in patients received standard PPI-based triple eradication therapy in Korea was 3.51% and the recrudescence rate 4.9% [11]. This result was similar to the mean annual reinfection rate (3.4%), calculated from the studies performed in developed countries [16]. The increase in antimicrobial resistance with the standard triple therapy has led to an increase of alternative therapy. However, there are few reports regarding the reinfection of *H. pylori* in patients received second-line therapy. In 2006, our group reported the annual reinfection rate after second-line therapy (EBMT) during 1996–2004, at 6.0% per patients-years in Seoul, Korea [12]. In the present long-term follow-up study for up to 90 months we investigated the reinfection rate of EBMT and MEA therapy, performed during 2003–2010 in Gyeonggi province near Seoul, and those rates are 4.45% for EBMT and 6.46% for MEA per year. When eradication has truly been successful, reinfection is associated with the risk of re-exposure to *H. pylori*. Relatively low reinfection rates might be related to the decrease in prevalence of *H. pylori* infection [21] and the recent improvement of sanitation conditions in Korea. In addition, when the reinfection rate of these two kinds of rescue therapy were compared, there was no significant difference (*p* = 0.43). Therefore, we suggest that reinfection cannot affect the choice of second-line treatment.

In our study, recrudescence rate in the EBMT group and MEA group was found to be 1.7% and 3.3%, which appears slightly lower than reported after initial eradication with standard PPI-based triple regimen (4.9%) [11]. This result might be related to the decreasing trend in eradication rate of standard triple therapy in Korea. That is, the eradication rate of per protocol (PP) analysis decreased up to 75.9% in 2006 [9]. Some studies reported that the recurrence of *H. pylori* infection more frequently occurred in patients treated with a low efficacy regimen than in those treated with a high efficacy regimen, as a result of recrudescence of the organism after temporary suppression, not elimination [14,16,22,23]. In the previous studies, the PP eradication rates were reported at 77.2% for the 7- and 93.6% for the 14 day EBMT regimen [24], and 83.8% for the 7-, 82.6% for the 10- and 79.9% for the 14 day MEA regimen [25]. Our lower recrudescence rate might be related to the higher efficacy of the EBMT and MEA regimens. In addition, there was no difference in the recrudescence rate between EBMT and MEA regimen (*p* = 0.67).

Limited information exists regarding risk factors for reinfection of *H. pylori*. Candidate risk factors include younger age [26,27], infection of close contacts [14,28], dental plaque [29,30], and contaminated endoscopic equipment [14,16,31]. Other studies did not identify any factors predictive of *H. pylori* reinfection [32-34]. In the previous study, we reported that male gender and low income were significantly associated with reinfection of *H. pylori* by multivariate analysis [11]. However, the current study did not identify any predictive factors concerning *H. pylori* reinfection in the EBMT group and MEA group.

This study is the first study with large sample size and a long-term follow-up period in the investigation of reinfection rate of *H. pylori* in patients who had been successfully treated with second-line therapy after an initial failure to eradicate *H. pylori*. However, our study has limitations. First, recrudescence cases could be included in the reinfection cases. Theoretically the fingerprinting should be performed for the differentiation of reinfected and recrudescence. However, we did not perform DNA analysis to identify the strains. In clinical practice, it is not easy to perform DNA analysis. Secondly, despite our efforts to enroll all patients, many patients dropped out from this study, and refused to receive *H. pylori* tests every year, especially when there was no gastrointestinal symptom.

## Conclusions

In summary, in Korea, the long-term reinfection rate of *H. pylori* stayed low in both bismuth-containing quadruple therapy and moxifloxacin-based triple therapy; thus reinfection cannot affect the choice of second-line treatment.

## Competing interests

The authors have no competing of interests to declare.

## Authors’ contributions

KN- designed the study and performed the major role of collecting patients; KMS- collected patients’ data and wrote the manuscript; KSE- collected patients’ data and was involved in editing the manuscript; JHJ- collected patients’ data and were involved in editing the manuscript; SCM- collected patients’ data and were involved in editing the manuscript; PYS- collected patients’ data and were involved in editing the manuscript; LDH- collected patients’ data and was involved in editing the manuscript. All authors read and approved the final manuscript.

## Pre-publication history

The pre-publication history for this paper can be accessed here:

http://www.biomedcentral.com/1471-230X/13/138/prepub
